# A Review of the Treatment of Herpes Simplex Virus-1 Encephalitis in Six Immunocompetent Patients

**DOI:** 10.7759/cureus.24129

**Published:** 2022-04-13

**Authors:** Edwin McCray, Tripp Atkinson, Molly Kearney, Eric Walker, Vipul Savaliya

**Affiliations:** 1 Internal Medicine, Campbell University School of Osteopathic Medicine, Buies Creek, USA; 2 Internal Medicine, Cape Fear Valley Medical Center, Fayetteville, USA; 3 Infectious Disease, Cape Fear Valley Medical Center, Fayetteville, USA

**Keywords:** herpes simplex virus type 1, hsv-1, hsv encephalitis, viral encephalitis, immuno-competent host, antiviral therapy

## Abstract

Introduction

The optimal treatment regimen for herpes simplex-1 (HSV-1) encephalitis is ill-defined. Current guidelines recommend the initiation of acyclovir in all suspected cases of encephalitis; however, there is limited research regarding the details of acyclovir treatment or the adjuvant use of corticosteroids. Specifically, there is a paucity of evidence-based guidelines detailing the optimal management of HSV-1 encephalitis in immunocompetent patients. In this study, we conducted a review of cases of immunocompetent patients with HSV-1 encephalitis to compare patterns in treatment and outcomes.

Methods

A review of the literature was performed using PubMed using the terms herpes encephalitis, HSV, herpes zoster, and immunocompetent to identify cases of HSV-1 encephalitis in immunocompetent patients. The results were screened for cases describing the treatment regimen of HSV-1 encephalitis-positive, immunocompetent patients.

Results

Six cases were identified. All six patients were treated with acyclovir with one patient receiving adjuvant corticosteroid therapy. Additionally, three patients were found to have acyclovir resistance and were transitioned to foscarnet. Eventually, one patient expired, two patients recovered with chronic morbidities of varying severity, and three patients made a full recovery.

Discussion

Inconsistencies in the patient's disease course, therapeutic regimen, and comorbidities could all play a role in the varying case outcomes. While the optimal timing and composition of therapies in HSV-1 encephalitis in immunocompetent patients are still unclear, it seems the timely administration of antiviral treatment remains essential. Further research is needed to optimize HSV-1 encephalitis therapeutic regimens and improve patient outcomes.

## Introduction

The optimal duration and composition of therapies for herpes simplex virus-1 (HSV-1) encephalitis are poorly understood. In HSV-1 encephalitis, the virus travels in a retrograde fashion along neuronal axons of the trigeminal nerve to the brain and often causes necrotic changes in the temporal lobe, which may occur independently of the host immune status [1,2]. Studies have demonstrated a mortality rate of 20-30% despite timely diagnosis and treatment [3-5]. The current standard of treatment is to initiate acyclovir therapy, empirically, on all patients with suspected encephalitis, and delaying treatment for greater than 48 hours is associated with worse outcomes [6-8]. The optimal duration of acyclovir therapy and any specific treatment differences based on patient-related factors have yet to be defined.

Previous studies on HSV-1 encephalitis have evaluated treatments outside of acyclovir therapy. Two clinical trials demonstrated that acyclovir was superior to vidarabine in reducing mortality in patients with HSV-1 encephalitis [4,9]. Additionally, the “Long Term Treatment of Herpes Simplex Encephalitis (HSE) With Valacyclovir” trial found that the administration of valacyclovir for 90 days after acyclovir therapy does not improve outcomes, as measured by neuropsychological testing with the Mattis Dementia Rating Scale (MDRS) at one year out [10]. Adjuvant treatment with corticosteroids is currently controversial. Since the brain damage from HSV-1 encephalitis is mostly immune-mediated, it has been theorized that the addition of corticosteroids may be beneficial in reducing cerebral edema and preventing autoimmune brain disease [8]. However, it is possible that suppressing the host immune response would promote viral replication [8]. To date, there have been no completed trials assessing the adjuvant use of corticosteroids with antivirals in the treatment of HSV-1 encephalitis, thus leaving the addition of corticosteroids a clinical decision by the treating provider. Currently, the Dex-Enceph Trial is underway, aiming to measure the effectiveness of acyclovir plus dexamethasone therapy in the treatment of HSV-1 encephalitis, with the primary outcome being the verbal memory score [6]. Speculation that intravenous immunoglobulin (IVIG) may be effective as a first-line treatment has been mentioned in the literature; however, no studies have been published on this [11].

Since there is a paucity of evidence-based treatment protocols in HSV-1 encephalitis, there is great variation in treatment regimens currently used. Additionally, there is a lack of evidence regarding the best practices specific to treating immunocompetent patients with HSV-1 encephalitis. We conducted a review of cases of immunocompetent patients with HSV-1 encephalitis to analyze the outcomes based on treatment-related factors. Identifying patterns related to outcomes may provide a foundation for future larger-scale studies in this patient population.

## Materials and methods

This is a retrospective review of published patients with laboratory-confirmed HSV-1 encephalitis with no known immunocompromising factors. The review was performed using a PubMed search using the terms herpes encephalitis, HSV, herpes zoster, and immunocompetent. PubMed is a large database containing over 32 million biomedical citations from MEDLINE, peer-reviewed medical journals, and online books. No restrictions were placed on publication date to ensure that pertinent cases were not unintentionally omitted. The resulting studies were screened for cases that describe the treatment regimen of immunocompetent patients with herpes simplex virus-1 encephalitis, including primary and adjuvant treatment. Cases of HSV-1 encephalitis infection, multiple virus associated encephalitis, or those including patients <18 years old were excluded. Additionally, cases that didn’t clearly denote the patient's immune status were excluded. 

Data were collected and used to compare treatment regimens and outcomes between cases. Patient age was the only demographic variable obtained. Cerebrospinal fluid (CSF) analysis data were extracted including glucose, protein, and white blood cell count. The use of acyclovir, valacyclovir, steroids, and “other” treatments were also recorded. Finally, the patients’ health-related outcomes were noted as per the last stated follow-up.

## Results

Six cases were retrieved, and the pertinent data were organized in table format (Table 1). In our review of cases of HSV-1 encephalitis in immunocompetent patients, only one patient received adjuvant corticosteroids, and no patients were treated with valacyclovir. Additionally, three patients were transitioned from acyclovir to foscarnet due to acyclovir resistance. One patient expired, three made a full recovery, and the remaining patients survived with chronic morbidities. 

**Table 1:** Data for cases included in the review -- denotes that these data were not reported or not applicable. WNL: within normal limits

Case	Authors & Year	Age	CSF glucose	CSF Protein	CSF WBC	Acyclovir	Steroids	Valacyclovir	Other	Outcome
1	Escobar-Valdivia et al., 2017 [12]	51	WNL	71 (H)	10	yes	no	no	Penicillin for concomitant neurosyphilis	survived
2	Bergmann et al., 2016 [13]	45	WNL	82 (H)	271 cell/ μL lymphocytes	yes	No	no	Foscarnet for acyclovir resistance	Survived with morbidities
3	Ye et al., 2019 [14]	43	--	51.6 mg/dL (H)	160 cells/μL	yes	yes	No	--	Survived with binocular blindness
4	Schulte et al., 2010 [15]	27	WNL	132 mg/dL (H)	292 cells/μL	yes	no	no	Foscarnet for acyclovir resistance	Survived with morbidities
5	Gain et al., 2003 [16]	40	WNL	120 mg/dL	25 cells/μL	yes	no	no	Foscarnet for acyclovir resistance	Survived with morbidities
6	Andreoletti et al., 2003 [17]	53	--	165 mg/dL	42 cells/μL	Yes	No	No	--	deceased

Escobar-Valdivia et al. describe a 51-year-old male patient that received acyclovir and survived without comorbidities [12]. He arrived at the emergency department unresponsive, tachycardic, and in respiratory acidosis. The patient was intubated and admitted to the intensive care unit for five days before being weaned off of mechanical respiratory support and transferred to the neurology service. At this time, CSF analysis showed 10 lymphocytes and elevated protein. The patient was started on intravenous penicillin and acyclovir for the treatment of neurosyphilis and HSV-1. The patient was alert and cooperative two weeks later and recovered without comorbidities. 

Bergman et al. describe the case of a 45-year-old female patient who survived with comorbidities following treatment with acyclovir and foscarnet [13]. The patient presented with aphasia and psychomotor slowing. CSF analysis showed elevated protein and lymphocytes. A polymerase chain reaction (PCR) test was positive for HSV-1; acyclovir 750 mg three times daily (TID) was initiated, which was subsequently increased to 1000 mg TID as the patient deteriorated. Following positive acyclovir resistance testing, foscarnet 60 mg TID was added with a dramatic improvement over the next two weeks. The patient was transferred to a neurological rehabilitation facility with residual neurological deficits. 

Ye. et al. conducted a case study on a 41-year-old patient treated with steroids and acyclovir, who survived with binocular blindness [14]. Prior to admission, the patient developed ophthalmodynia, conjunctival redness, and blurred vision in the left eye. Despite treatment with levofloxacin, the vision of his left eye further deteriorated, and the visual acuity in his right eye began to worsen. The patient was diagnosed with bilateral uveitis and prescribed intravenous methylprednisolone for three days. Five days later, the patient developed a severe headache followed by a sudden three-minute sustained, generalized tonic-clonic seizure, for which he was admitted to the emergency department. CSF analysis showed pleocytosis and increased protein. Acyclovir treatment for suspected viral encephalopathy was initiated. Subsequent PCR testing detected HSV-1 DNA in the patient’s vitreous humor. The patient was discharged after 21 days of acyclovir treatment with continued bilateral blindness. 

The case study conducted by Schulte et al. describes a 27-year-old woman treated with acyclovir and foscarnet who survived with morbidities [15]. The patient presented with a four-day history of right-sided headaches, personality change, fever, and left-sided hemiparesis. Her CSF analysis showed pleocytosis and increased protein levels, with PCR confirming HSV-1 infection. Despite the initiation of acyclovir treatment, the patient's status continued to deteriorate over the following days. A second lumbar puncture performed six days after admission revealed an increase in pleocytosis and protein levels compared to her results upon admission. The medical team initiated foscarnet treatment due to clinical suspicion of acyclovir-resistant HSV-1. After 14 days of foscarnet treatment and 19 days of acyclovir treatment, the patient was transferred to a rehabilitation facility. 

Gain et al. describe the case of a 40-year-old man who survived with morbidities following treatment with acyclovir and foscarnet [16]. The patient was admitted to the hospital following the sudden onset of febrile convulsions. Over the next two days, the patient's neurological condition worsened from disorientation to a deep coma. CSF analysis found increased lymphocytes and protein. HSV-1 infection was confirmed using PCR, and acyclovir treatment was initiated. After seven days of persistent altered mental status, acyclovir-resistant HSV was assumed, and the team began foscarnet treatment. After three weeks, the patient was discharged with continued anterograde amnesia, anosmia, and slowed cognitive function. Forty-five days after the initial admission, the patient was readmitted with a rapid decrease in visual acuity. The patient once again received foscarnet treatment for one month and survived with loss of visual function. 

Andreoletti et al. describe the case of a 53-year-old patient who expired despite acyclovir treatment [17]. The patient presented with febrile encephalitis syndrome for which he was admitted. CSF analysis revealed elevated leukocytes and protein. Acyclovir treatment was initiated 24 hours after admission. The patient continued to deteriorate while receiving acyclovir treatment and expired after 15 days. 

## Discussion

Worldwide, sporadic acute viral encephalitis is most frequently caused by HSV; HSV-1 is responsible for approximately 90%, with immunocompromised patients generally being affected by HSV-2 [6,8]. Currently, the incidence of HSE is estimated to be two to four per 1,000,000 in the United States, with up to 55% of healthy, middle-aged adults showing seropositivity for HSV-1 [7,8]. 

The most common manifestation of HSE includes nausea, vomiting, headaches, focal deficits, confusion, and seizures [8,18]. HSV generally infects the temporal lobe, trigeminal nerve, and the brainstem, potentially leading to amnesia, neurogenic pain, and upward gaze palsies [18]. Symptoms usually develop rapidly and can lead to decreased consciousness leaving about a third of patients requiring ICU admission and intubation [8]. Immunocompromised patients can develop venous thromboembolism, inappropriate antidiuretic hormone secretion, and secondary infections [8]. The most serious complication of HSE is increased intracranial pressure that can lead to uncal and/or transtentorial herniation [8]. Kiyani et al. recently examined the mortality of patients with viral encephalitis, as well as the cost associated with caring for these patients. In a retrospective examination of patients in the MarketScan database between 2008 and 2015, over 8,000 patients were diagnosed with viral encephalitis; 38.3% were found to have HSV-1 encephalitis [18]. Interestingly, 29.2% of those with HSE were in the southern United States, demonstrating a possible regionality association [18]. When compared to patients diagnosed with non-herpetic encephalitis, those with uncal and/or transtentorial herniation had higher median healthcare costs at one year ($27,845 and $37,403 respectively) and a higher mortality rate at five years (5.8% and 8.9% respectively) [18]. In general, immunocompromised patients have been shown to have more extensive brain involvement, therefore presenting with lesser focal neurologic symptoms [6]. These patients were also found to have significantly high mortality compared to immunocompetent patients diagnosed with HSV-1 encephalitis (35.7% and 6.7%, respectively) [6]. 

As stated previously, outcomes of the six HSV-1 encephalitis cases included in our review varied drastically. One patient expired, one made a full recovery, and the remaining patients survived with chronic morbidities. Outcome discrepancies could potentially be explained by variations in patient disease course, treatment regimen, or prior comorbidities. 

In case one, the patient was treated with acyclovir without corticosteroids. Penicillin and sodium valproate were administered in addition to acyclovir for treatment of concomitant neurosyphilis. The patient went on to make a full recovery despite being the only multi-pathogen infection in our review. 

The patients surviving with residual morbidities received a mixture of treatment regimens. Three patients received acyclovir followed by foscarnet without corticosteroids, and one patient received acyclovir and corticosteroids without foscarnet. Foscarnet was administered five to seven days following acyclovir in these cases for management of acyclovir-resistant HSV. Acyclovir-resistant HSV has a known and consistent prevalence of about 0.3% in immunocompetent patients [19, 20]. This delay in initiating a definitive treatment could explain the poorer outcomes observed with this treatment. The patient treated with adjuvant corticosteroids received methylprednisolone five days before beginning acyclovir due to an early misdiagnosis. Although the patient survived, it is unclear if the accidental addition of methylprednisolone affected the outcome. 

The only patient to expire received acyclovir alone. Like some patients described earlier, the outcome may have been associated with an acyclovir-resistant strain of HSV. Not administering a stain-appropriate antiviral treatment could explain the patient's expiration, but without the HSV strain's genome sequencing or anti-viral susceptibility data, this is only speculation. 

Although no conclusive statements can be made regarding the appropriate treatment for HSV-1 encephalitis in immunocompetent patients, it seems clear that timely administration of antiviral medications is an integral part of the management strategy. Since these cases contain a small sample size, no control, and limited data, the outcomes are not necessarily applicable to the general population of immunocompetent patients with HSV-1 encephalitis. However, the financial and health-related quality of life burden associated with HSV-1 encephalitis is significant and warrants further investigation, with the goal of developing an evidence-based treatment algorithm that may consider various antivirals with or without corticosteroids, or immunotherapy. 

## Conclusions

The standard treatment of HSV-1 encephalitis is IV acyclovir, as PO acyclovir and valacyclovir require multiple doses per day. Currently, there are no hard treatment guidelines addressing IV acyclovir with adjuvant corticosteroids as a treatment for HSV-1 encephalitis. The current standard of treatment is to begin acyclovir therapy on all patients with suspected viral encephalitis. Since a definitive treatment regimen has yet to be established, clinicians must use their professional judgment when managing this patient population. Prior studies have demonstrated that timely antiviral treatment for HSV-1 encephalitis remains critical; however, further investigation into the optimal duration and composition of HSV-1 encephalitis therapy is warranted to optimize patient outcomes. Corticosteroids and immunotherapy may play a role in improving health-related outcomes, along with a further understanding of indications for foscarnet therapy.
